# Caudal ropivacaine–clonidine: A better post-operative analgesic approach

**DOI:** 10.4103/0019-5049.65368

**Published:** 2010

**Authors:** Sukhminder Jit Singh Bajwa, Jasbir Kaur, Sukhwinder Kaur Bajwa, Geetika Bakshi, Kanwalpreet Singh, Aparajita Panda

**Affiliations:** Department of Anaesthesiology and Intensive Care, Gian Sagar Medical College & Hospital, Ram Nagar, Banur, Punjab, India

**Keywords:** Caudal block, clonidine, ropivacaine

## Abstract

The aim was to determine qualitative and quantitative aspects of caudal block, haemodynamic effects, and post-operative pain relief of ropivacaine 0.25% versus ropivacaine 0.25% with clonidine for lower abdominal surgeries in paediatric patients. A double-blind study was conducted among 44 paediatric patients in the Department of Anaesthesiology and Intensive Care of our institute. A total of 44 ASA-I paediatric patients between the ages of 1 and 9 years, scheduled for elective hernia surgery, were enrolled in this randomised double-blind study. The caudal block was administered with ropivacaine 0.25% (Group I) and ropivacaine 0.25% and clonidine 2 µg/kg (Group II) after induction with general anaesthesia. Haemodynamic parameters were observed before, during and after the surgical procedure. Post-operative analgesic duration, total dose of rescue analgesia, pain scores and any side effects were looked for and recorded. All the results were tabulated and analysed statistically. The variables in the two groups were compared using the non-parametric tests. For all statistical analyses, the level of significance was *P* < 0.05. Forty-four patients were enrolled in this study and their data were subjected to statistical analysis: 22 patients in both the groups were comparable with regard to demographic data, haemodynamic parameters and other vitals and were statistically non-significant (*P*>0.05). The duration of analgesia was significantly prolonged in Group II (*P*<0.05). The dose requirement for post-operative pain relief was also significantly lesser in Group II. The incidences of side effects were almost comparable and non-significant. A caudal block with 0.25% of isobaric ropivacaine combined with 2 µg/kg of clonidine provides efficient analgesia intra-operatively and prolonged duration of analgesia post-operatively.

## INTRODUCTION

In paediatric regional analgesia, caudal epidural technique is one of the most popular, reliable, safe and easy methods to administer and is therefore the commonly performed procedure for intra-operative and post-operative analgesia especially for sub-umbilical surgeries in young children. One of the main drawbacks of this technique is the short duration of analgesia even with the use of long-acting local anaesthetics like bupivacaine and ropivacaine.[1] The success of achieving prolonged duration of analgesia by the addition of an adjuvant to these local anaesthetics has kept the interest of anaesthesiologists alive for the search of a new adjuvant. The recent literature has cited the double-caudal technique whereby topped up caudal solution is injected at the end of surgery but its popularity is still limited on the grounds of toxicity due to large volume of drugs injected.[2]

Ropivacaine has been extensively used for regional anaesthesia in adults and older children[3] and has been used safely even in the younger age group as well for caudal epidural analgesia.[4–6] The lower incidence of cardiovascular side effects and neurotoxicity as well as the ability to produce lesser motor blockade has made the ropivacaine a safer choice as compared to bupivacaine for caudal epidural anaesthesia especially for day care surgeries.[3,7] A higher concentration of ropivacaine 0.5% (0.75 ml/kg) is associated with a prolonged duration of analgesia as compared to 0.25% ropivacaine but at this level plasma levels are high and can cause early signs of toxicity in children along with an increased motor blockade.[8]

Clonidine is an alpha-2 adrenoceptor agonist which was widely used as an antihypertensive in 70s and 80s and presently it has been increasingly used for sedation, premedication and as an adjuvant analgesic.[9,10] The post-operative analgesia increased significantly with the addition of clonidine to caudal bupivacaine 0.25% in children aged 1–7 years who underwent sub-umbilical general, urological and orthopaedic surgery as compared to plain bupivacaine 0.25%.[11,12]

The addition of clonidine as an adjuvant has allowed the use of lower concentration of the local anaesthetic for achieving the same level of anaesthesia but with a prolonged duration of analgesia which increases the margin of safety and reduces the incidence of unwanted motor blockades.[11–13] With these facts in mind we undertook the study to compare the analgesic properties of 0.25% ropivacaine with the addition of clonidine (2 *µ*g/kg) to that of ropivacaine 0.25% following caudal administration in children.

## METHODS

After the approval from the institutional ethics committee, written informed consent of the parents was obtained. We enrolled 44 ASA-I children, 1–9 years of age, scheduled for elective lower abdominal surgery (hernia surgery) for this study. The study design was randomized and double blind; patients were randomly allocated according to a computer-generated randomisation. The sample size represented the population of 7–8 lakh in the vicinity of 35–40 km radius which the institution caters to. Exclusion criteria consisted of local infection at the caudal region, bleeding diathesis, pre-existing neurologic or obvious spinal diseases, and any congenital anomaly of the lower back.

Patients were given oral midazolam (0.3 mg/kg) as premedication approximately 1 h prior to arrival in the pre-operative room. All the baseline parameters like heart rate (HR), mean arterial pressure (MAP) and peripheral oxygen saturation (SpO_2_) were observed and recorded. A good IV access was secured; an induction of anaesthesia was achieved with sevoflurane 8% and 60% nitrous oxide (N_2_O) in oxygen. All the patients were paralysed with injection atracurium 0.5 mg/kg and were intubated with an appropriate sized endotracheal tube. After securing the endotracheal tube, patients were turned to the left lateral position for the administration of caudal anaesthesia which was achieved with 23 gauge intravenous needle under all aseptic conditions and the patients were turned supine immediately after the injection. Group I (*n* = 22) received 0.25% ropivacaine, 0.5 ml/kg, while patients in Group II (*n* = 22) received 0.25% ropivacaine, 0.5 ml/kg, with an addition of 2 *µ*g/kg clonidine *via* the caudal route with a total volume being constant at 0.5 ml/kg in both the study groups. The pin-prick method was used to assess the level of sensory anaesthesia and the variation in HR was chosen as the response variable to confirm the dermatomal level which was attained up to T-8 to T-9 level in almost all of the patients.

The syringes for the study solutions were prepared by a senior resident of the anaesthesiology department who was given written protocols for drug preparation and was unaware of the patients and operation theatre team. Anaesthesia was subsequently maintained with sevoflurane (2–3%)–oxygen–N_2_ O and patients were mechanically ventilated with the Jackson–Rees circuit. Haemodynamic parameters, respiratory rate (RR), end-tidal CO_2_ concentration (EtCO_2_) and peripheral oxygen saturation (SpO_2_) were recorded before induction, after induction, after intubation and then after caudal anaesthesia and at 10-min interval thereafter. Any increase in MAP or HR of more than 15% from the baseline observations and values during the surgical procedure was taken out of the purview of haemodynamic stability attributable to caudal analgesia. An increase in HR or MAP within 10–15 min of the start of the surgical procedure was adjudged as failure of caudal anaesthesia, and rescue analgesia in the form of fentanyl was administered (2 *µ*g/kg). Intravenous fluids were administered according to body weight and the fasting status in the form of Isolyte-P solution.

At the end of the surgical procedure all the anaesthetic gasses were turned off and the patients were extubated in a fully awake condition. MAP, HR, SpO_2_, pain and sedation scores (opening of eyes: 3 = spontaneously, 2 = to verbal command, 1 = to physical shaking, 0 = not arousable) were recorded at a 10-min interval after extubation and thereafter at intervals of 1, 2, 4, 6, 8, 12, 18 and 24 h. A modified objective pain scale (OPS) was employed to assess the post-operative pain and duration of analgesia which was based on behavioural objectives that included crying, facial expressions, position of legs, position of torso and generalized motor restlessness. A score of 0 was considered as excellent analgesia while a score of 10 signifies completely ineffective analgesia. Children who had a pain score of more than 4 were administered 15 mg/kg of oral syrup of paracetamol. The total amount of the analgesic dose and any complication or side effects were looked for and recorded. All the patients were observed for next 24 h in the special rooms and the recordings of all parameters were done by a senior resident of Anaesthesiology and a well-trained staff. All the observations were recorded half hourly for the first 6 h and thereafter hourly till the next 18 h. The patients were discharged the next day and the parents were given phone numbers to contact in the case of any untoward incident. During the follow-up after 1 week, none of the parents complained of any side effects or untoward incident. The data from the two groups were subjected to statistical analyses with the help of non-parametric tests and *P* values <0.05 were considered statistically significant. Data are presented mainly as arithmetic means and standard deviations.

## RESULTS

We enrolled 44 children (22 children in each group) in our study profile. No difference could be detected from the data of 44 children regarding the patient demographics.

The demographic profile of the patients in group I and group II was comparable with regards to age, weight and height and on statistical analysis no significant difference was found as is clearly evident from the Table 1.

**Table 1:** Demographic data in both the groups

Demographic characteristics	Group I	Group II
Age (years)	3.1 ± 1.68 (1–9)	3.4 ± 1.42 (1–9)
Weight (kg)	13.12 ± 7.86	13.92 ± 6.16
Height (cm)	96 ± 6	97 ± 3
Gender (M/F)	21/1	20/2

Table 2 conveys the comparison of various vital parameters of the patients of both the groups. Intra-operative HR, NIBP, ETCO_2_ and SpO_2_ showed no statistical significant difference between the two groups (*P*>0.05). There was no statistically significant difference between the two groups regarding the duration of surgery or time to extubation from cessation of anaesthesia (*P* > 0.05). No significant hypotension or bradycardia was observed in any patient. SpO_2_ (>97%) was always within the clinically acceptable range in both the groups throughout the procedure (*P* > 0.05).

**Table 2:** Vital parameters of both the groups

	Group I	Group II	*P* value
Pre-op. HR	113.78 ± 12.54	117.32 ± 11.22	0.68
Intra-op. HR	109.16 ± 7.94	107.20 ± 8.02	0.74
Post-op. HR	97.18 ± 4.98	94.46 ± 7.38	0.67
Pre-op. MAP	71.94 ± 9.78	72.26 ± 12.14	0.80
Intra-op. MAP	65.74 ± 8.32	63 ± 8.16	0.66
Post-op. MAP	69.86 ± 9.92	68.08 ± 11.648	0.79
Intra-op. SpO_2_	97 ± 2.48	97 ± 2.62	0.95
Post-op. SpO_2_	96 ± 3.36	96 ± 2.76	0.86
Duration of surgery (min)	49.64 ± 12.88	48.14 ± 12.32	0.44
Time to extubation (min)	5.12 ± 2.28	5.48 ± 2.66	0.48

It is quite clear from the Table 3 that the first analgesic requirement time was statistically prolonged in Group II (13.4 ± 3.4 h) when compared with Group I (8.5 ± 3.4 h) (*P* < 0.05). Total analgesic consumption was statistically higher in Group I (172 ± 80 mg) when compared with Group II (96 ± 72 mg) (*P* < 0.05). Six children required paracetamol administration once and 1 child required it twice in Group II as compared to 11 children requiring it once and 8 children requiring paracetamol twice in Group I (*P* < 0.05). Fifteen children in Group II and three children in Group I required no additional pain medication during the first 24-h study period, which was statistically significant (*P* < 0.05).

**Table 3:** Drug characteristics in both the groups

Characteristics	Group I	Group II	*P* value
Mean sedation scores (h)	2.68 ± 0.56	2.86 ± 0.52	0.71
Duration of analgesia (h)	8.5 ± 3.4	13.4 ± 3.4	<0.05
Mean OPS score	3.72 ± 0.42	3.58 ± 0.40	0.59
Total analgesic dose	172 ± 80	96 ± 72	<0.05

**Figure 1:**
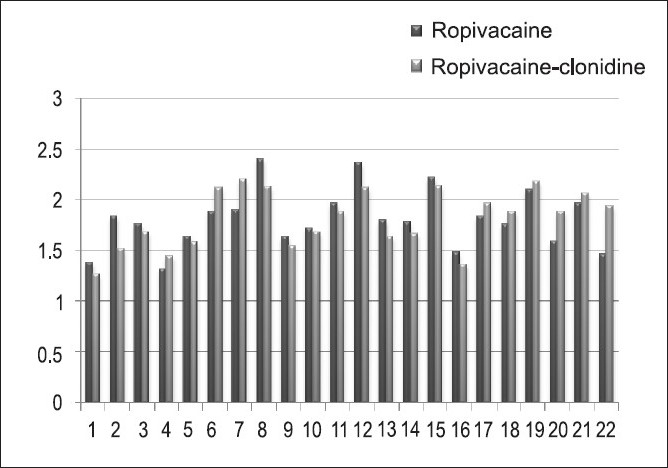
The comparison of mean OPS scores in Group I and Group II

As is evident from the Figure 1, the mean OPS score in both the groups were comparable and no significant difference between both the groups can be made out from the above bar diagrm.

The post-operative sedation scores showed no statistically significant difference (*P* > 0.05) which is clearly evident from the description in Table 4. No motor impairment was seen in either group on awakening and during the next 24-h period.

**Table 4:** The comparison of sedation scores of patients in both the groups

Sedation score	Group I	Group II
0	None	None
1	2	2
2	6	7
3	14	13

Table 5 depicts the comparative incidence of side effects in both the groups. Two patients in Group II had episode of vomiting as compared to one patient in Group I which was not significant on statistical analysis (*P*>0.05). No any other untoward side effects were observed in either of the groups.

**Table 5:** The side effects in both the groups

Side effects	Group I	Group II
Vomiting	1	2
Urinary retention	0	0
Respiratory depression	0	0
Miscellaneous	None	None

## DISCUSSION

Clonidine is being increasingly used nowadays for potentiating the analgesic action of various local anaesthetics administered regionally. The main interest of our study was to evaluate the efficacy of caudal clonidine when combined with the 0.25% solution of ropivacaine. Despite the hernia surgery being a day care procedure we decided to keep the patients in special rooms in the hospital which were fully furnished to give a ‘feeling at home’ to the patients and to their respective parents. This was mainly done to obviate any bias or error in the findings of the study. The main finding of the present study is that a caudal bolus injection of a combination of ropivacaine 0.25% with clonidine 2 *µ*g/kg provides better postoperative analgesia compared to ropivacaine 0.25% alone.

The quest for finding the ideal combination of drugs for caudal anaesthesia in children is never-ending but the efforts to use the relatively safer drugs and that too in lower concentration are growing day by day. Ropivacaine is one such drug that appears to be associated with a greater safety margin and reduced systemic toxicity although such toxicity has been reported in adults following various regional anaesthetic techniques.[14,15] Ropivacaine when used in a reduced concentration below 0.2% in children is hardly effective and that is the reason we adhered to a concentration of 0.25%.[16] Clonidine produces analgesia via a non-opioid mechanism.[17] Klimscha *et al*. had studied the effectiveness of caudal clonidine in potentiating the post-operative analgesic effect and found that in small children with a mean age of 3 years who underwent an elective day care surgery for hernia operations, the addition of clonidine 1–2 *µ*g/kg to bupivacaine 0.25% significantly prolonged the median duration of analgesia and reduced the total dose of post-operative analgesics compared with bupivacaine alone or bupivacaine plus epinephrine 5 *µ*g/ml (*P*<0.05).[18] The findings of our study are almost similar with the observations of Klimscha *et al*. as post-operative analgesia was significantly prolonged in the patients receiving clonidine as an adjuvant to ropivacaine.

Clonidine given by the neuraxial route decreases the impulse generation by preganglionic sympathetic nerves. Similarly, the dominance of the parasympathetic nervous system results in an increased vagal tone which causes bradycardia.[19] We did observe a fall in MAP and a 3–5% decrease in the heart rate in Group II patients but it got stabilized to normal within 20–30 min of the caudal injection.

Clonidine causes dose-dependent post-operative sedation in children as demonstrated by Lee and his colleagues in their study on adding 2 *µ*g/kg clonidine to caudal bupivacaine.[12] In our study, the difference in sedation scores was not statistically significant as the all patients were easily arousable in both the groups which is consistent with the findings of other studies.[20]

Ropivacaine produces a lesser post-operative motor blockade as compared to bupivacaine when used in a lower concentration.[21,22] There was no apparent motor deficit in our patients probably due to the lower concentration of ropivacaine used. The power analysis of the study was done and we found out the value to be 87.6%.

## CONCLUSIONS

We conclude that a single caudal injection of clonidine (2 *µ*g/kg) added to ropivacaine 0.25% offers an advantage over 0.25% ropivacaine alone for post-operative pain relief in children undergoing lower abdominal surgery, without increasing the incidence of adverse effects.
